# Supplementary material for: IKKε and TBK1 prevent RIPK1 dependent and independent inflammation
Source: Nat Commun. 2024 Jan 2;15:130. doi: 10.1038/s41467-023-44372-y (PMC10761900; doi:10.1038/s41467-023-44372-y)
Supplement: Supplementary file 1 — Supplementary Information [file 41467_2023_44372_MOESM1_ESM.pdf]

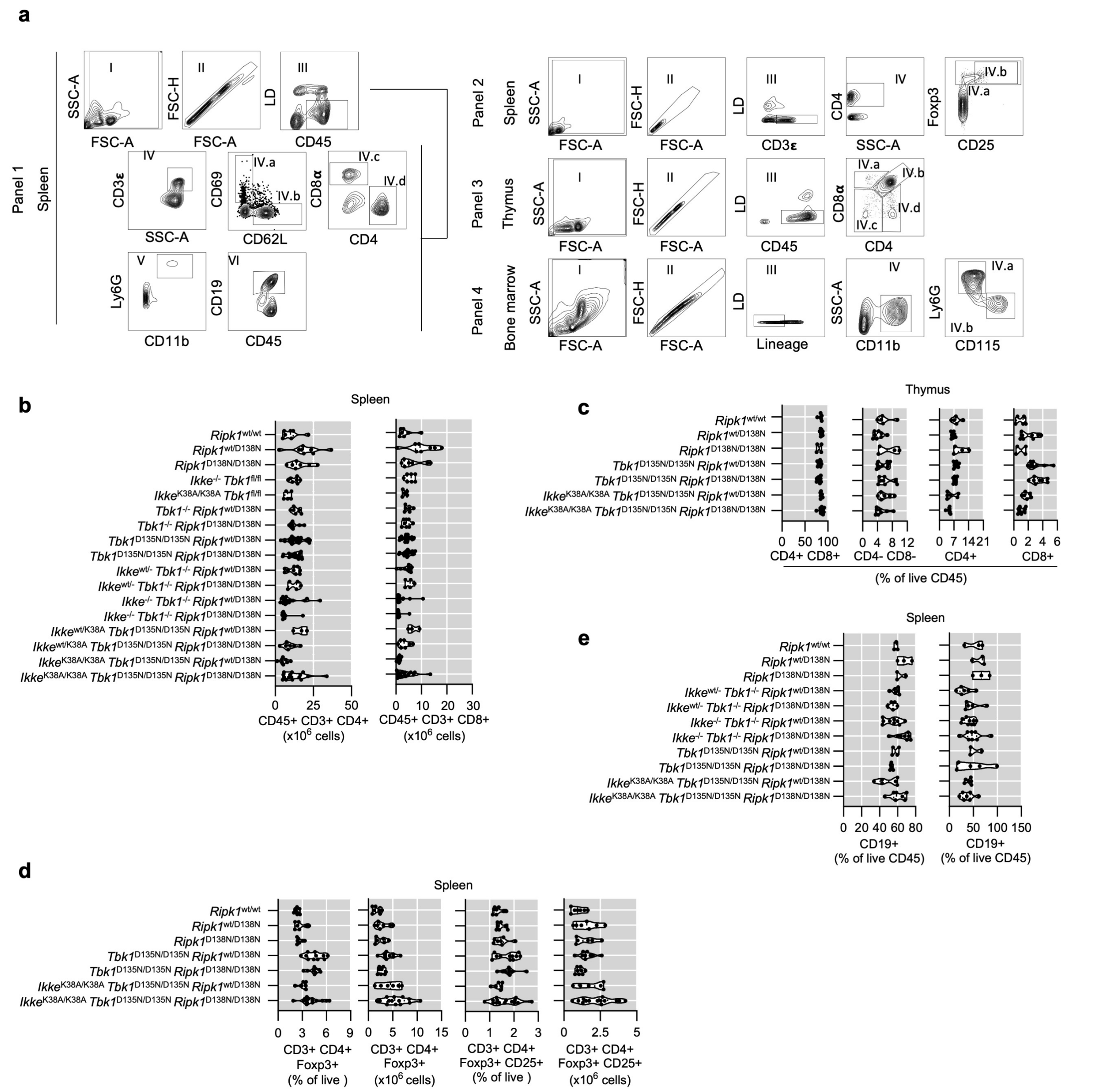

Supplementary Figure 1. Flow cytometric assessment of immune cell populations in mice with combined deficiency or kinase inhibition of TBK1 and IKKε.

(**a**) Panel 1: Flow cytometry gating strategy for sequential exclusion of debris (I), doublets (II) and dead cells and CD45- cells (III) to gate on activated T cells (CD45+CD3+CD69+) and naïve T cells (CD45+CD3+CD62L+) (IV.a), T cells (CD45+CD3+CD4+ and CD45+CD3+CD8+) (IV.b), neutrophils (CD45+CD11b+Ly6G+) (V), and B cells (CD45+CD19+) (VI) in spleen. Panel 2: Flow cytometry gating strategy for sequential exclusion of debris (I) doublets (II) and dead cells (III) to gate on CD3ε+ CD4+ (IV), CD3ε+ CD4+ Foxp3 (IVa), and CD3ε+ CD4+ Foxp3+ CD25+ (IVb). Panel 3: Flow cytometry gating strategy for sequential exclusion of debris (I), doublets (II) and dead cells and CD45- cells (III) to gate on single positive (CD45+CD4+CD8- and CD45+CD4-CD8+) (IV.a and IV.d), double positive (CD45+CD4+CD8+) (IV.b), and double negative (CD45+CD4-CD8-) (IV.c) in thymus. Panel 4- Flow cytometry gating strategy for sequential exclusion of debris (I), doublets (II) and dead cells and CD3ε+ CD19+ NK1.1+ B220+ cells (III) to gate on CD11b+ Ly6G+ (neutrophils) (IV.a) and CD11b+ CD115+ (monocytes) (IV.b) in bone marrow. (**b**) Graphs showing counts of CD3+ CD4+ and CD3+ CD8+ cells amongst live splenocytes of 8-13 week-old mice with indicated genotypes, assayed with flow cytometry. (**c**) Graphs showing percentage of CD4+ CD8+, CD4- CD8-, CD4+ CD8-, and CD4- CD8+ amongst live and CD45+ thymocytes of 8-13 week-old mice with indicated genotypes.

(**d**) Graphs showing percentage and cell counts of CD3+ CD4+ Foxp3+ and CD3+ CD4+ Foxp3+ CD25+ cells amongst live splenocytes of 8-13 week-old mice with indicated genotypes. (**e**) Graphs showing percentage and cell counts of spleen residing CD19+ (B cells) amongst total live cells (left) or live CD45+ cells (right) of 8-13 week-old mice with indicated genotypes, assayed by flow cytometry. Each dot represents one mouse. $^{*}p < 0.05$, $^{**}p < 0.01$, $^{***}p < 0.005$, $^{****}p < 0.0001$ (one-way ANOVA t-test with post-hoc multiple test). Violin plots show the median of biological replicates and interquartile range of data. Source data for **b-e** are provided as a source data file.

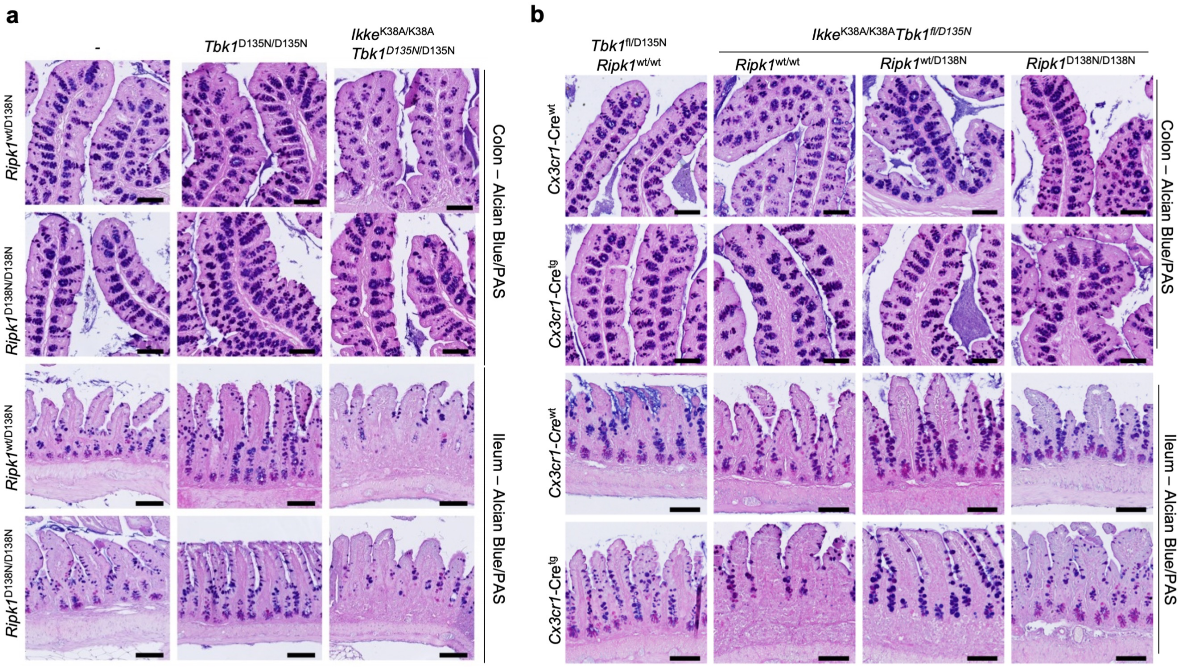

Supplementary Figure 2. Combined deficiency or kinase activity inhibition of TBK1 and IKKε systemically or specifically in myeloid cells causes loss of secretory cells in the intestine.
(**a, b**) Representative images of colon and small intestine sections of 8-13 week-old mice with the indicated genotypes stained with Alcian blue and periodic acid Schiff staining. Scale bars, 100µm.

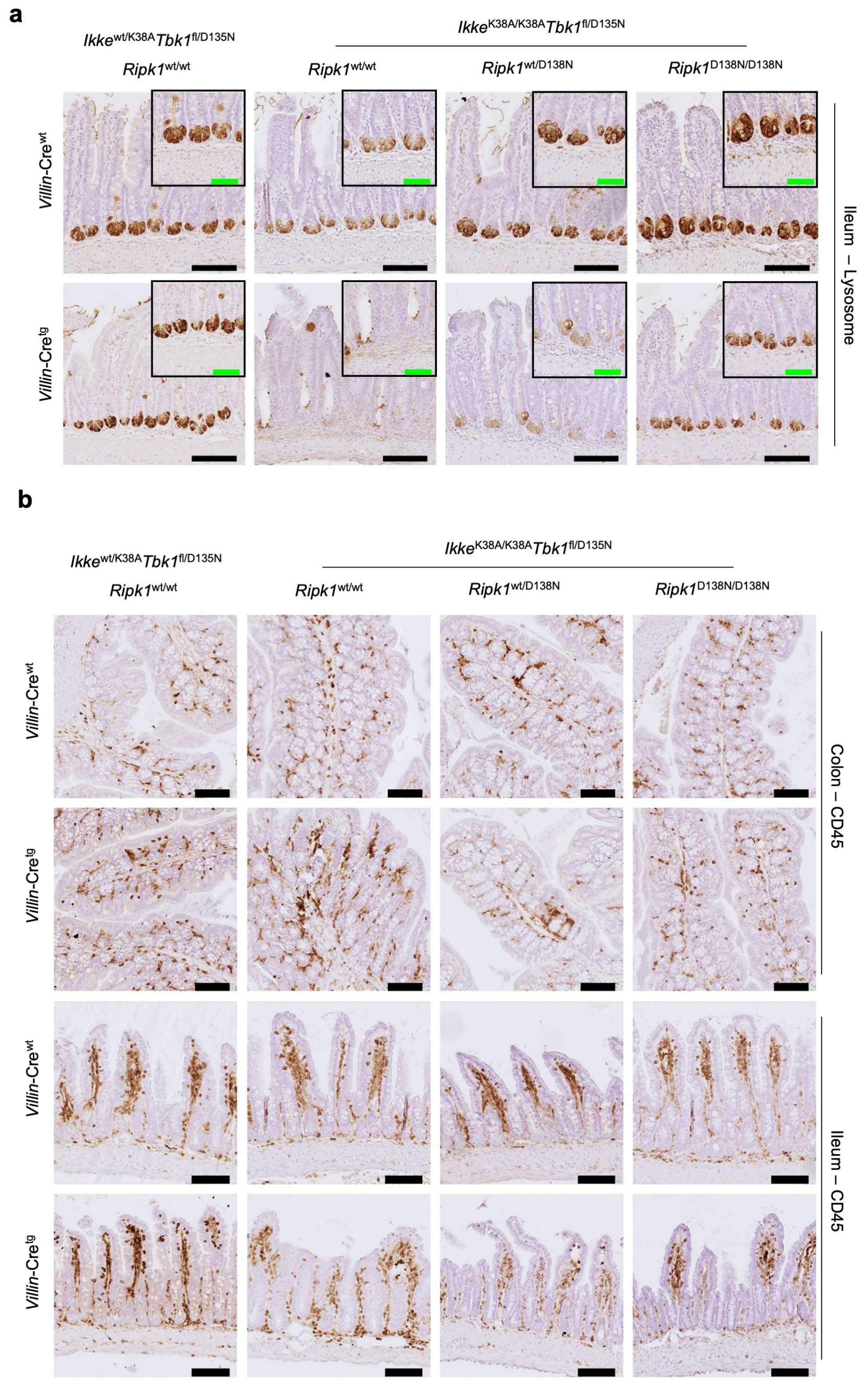

Supplementary Figure 3. Combined inhibition of TBK1 and IKKε kinase activities specifically in IECs causes RIPK1-dependent loss of Paneth cells and immune cell infiltration in the intestine. (**a, b**) Representative images of colon and small intestine sections of 5-6 week-old mice with the indicated genotypes immunostained for lysozyme (**a**) or CD45 (**b**). Scale bars, 100µm.

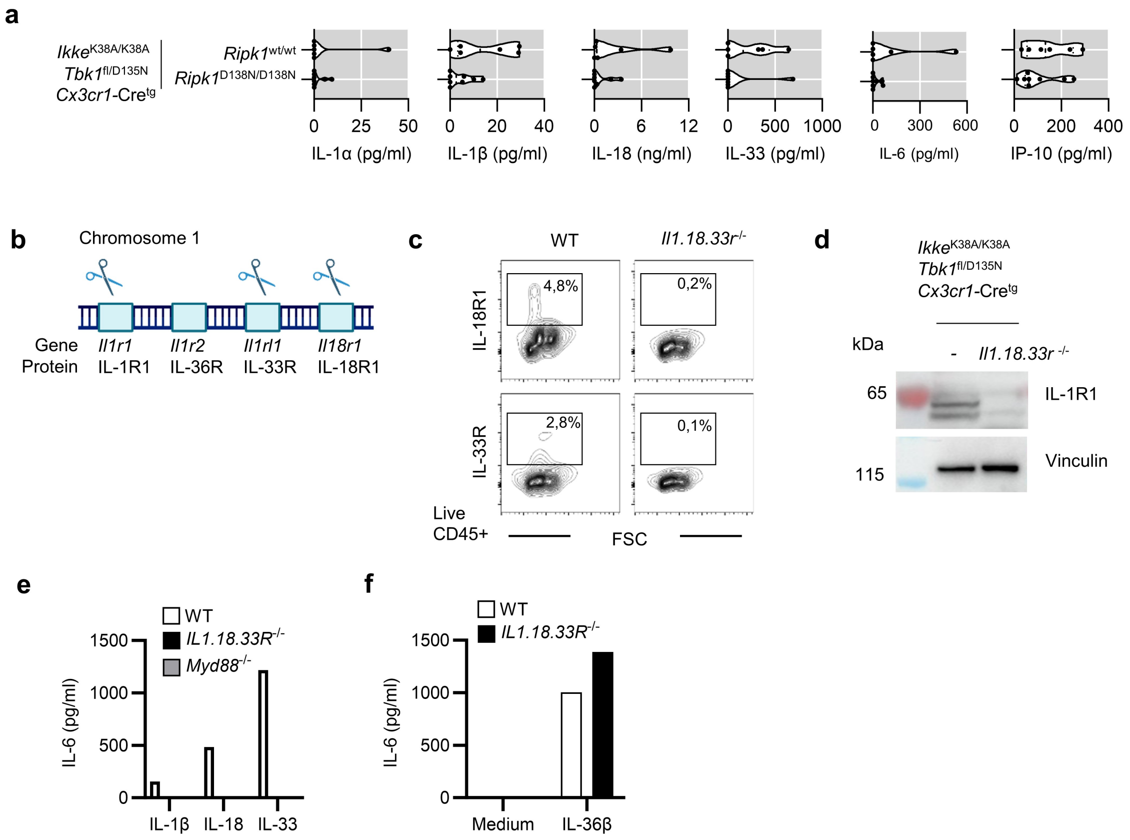

Supplementary Figure 4. Generation and validation of mice with combined deficiency in IL-1R, IL-18R, and IL-33R (*Il1.18.33r*$^{-/-}$).

(**a**) Graphs showing the levels of IL-1α, IL1β, IL-18, IL-33, IL-6 and IP-10 in the serum of *Ikke*$^{K38A/K38A}$ *Tbk1*$^{fl/D135N}$ *Ripk1*$^{wt/wt}$ *Cx3cr1-Cre*$^{wt/wt}$ and *Ikke*$^{K38A/K38A}$ *Tbk1*$^{fl/D135N}$ *Ripk1*$^{D138N/D138N}$ *Cx3cr1-Cre*$^{wt/tg}$ mice. (**b**) Schematic depicting the CRISPR/Cas9-mediated targeting of the *Il1r1*, *Ilrl1*, and *Il18r1* genes located in chromosome 1. (**c**) Graphs showing flow cytometric analysis of PE conjugated anti-IL-18R1 or -IL-33R immunostained live CD45+ primary bone marrow cells from indicated genotypes. (**d**) Immunoblot analysis with the indicated antibodies of whole cell lysates from the primary bone marrow cells from mice with the indicated genotypes. (**e**) Graph showing IL-6 levels in the supernatant of 1x10^{6} primary bone marrow cells from WT, *Il1.18.33r*$^{-/-}$, and *Myd88*$^{-/-}$ mice treated with 10ng/ml IL-1β, IL-18, or IL-33 for 24 hours. (**f**) Graph showing IL-6 levels in the supernatant of 5x10^{6} primary bone marrow cells from WT and *Il1.18.33r*$^{-/-}$ mice treated with 100ng/ml IL-36Rβ for 24 hours. Violin plots show the median of biological replicates and interquartile range of data. Source data for **c-f** are provided as a source data file.

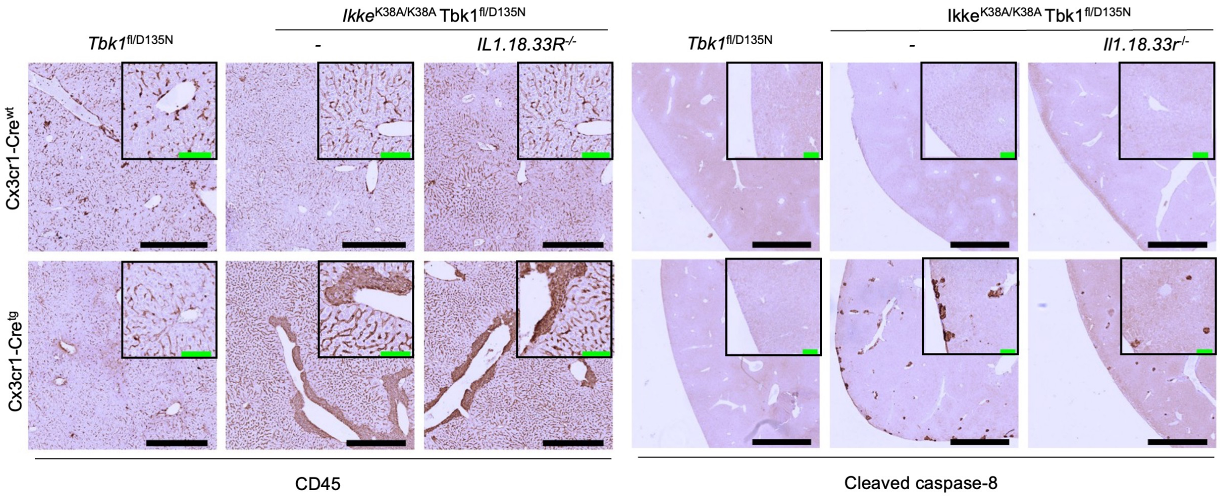

Supplementary Figure 5. Combined deficiency in IL-1R, IL-18R, and IL-33R ameliorates cell death and inflammation in the liver of *Ikke*$^{K38A/K38A}$ *Tbk1*$^{D135N/D135N}$ *Cx3cr-1Cre*$^{wt/tg}$ mice.
Representative images of liver sections from 8-13 weeks-old mice with the indicated genotypes immunostained for CD45 and cleaved caspase 8. Scale bar, 1mm (black) and 100μm (green).
