# Supplementary material for: IKKε and TBK1 prevent RIPK1 dependent and independent inflammation
Source: Nat Commun. 2024 Jan 2;15:130. doi: 10.1038/s41467-023-44372-y (PMC10761900; doi:10.1038/s41467-023-44372-y)
Supplement: Supplementary file 3 — Reporting Summary [file 41467_2023_44372_MOESM3_ESM.pdf]

nature portfolio

Corresponding author(s): Manolis Pasparakis

Last updated by author(s): Nov 14, 2023

Reporting Summary

Nature Portfolio wishes to improve the reproducibility of the work that we publish. This form provides structure for consistency and transparency in reporting. For further information on Nature Portfolio policies, see our Editorial Policies and the Editorial Policy Checklist.

Statistics

For all statistical analyses, confirm that the following items are present in the figure legend, table legend, main text, or Methods section.

n/a	Confirmed	
☐	☒	The exact sample size (n) for each experimental group/condition, given as a discrete number and unit of measurement
☐	☒	A statement on whether measurements were taken from distinct samples or whether the same sample was measured repeatedly
☐	☒	The statistical test(s) used AND whether they are one- or two-sided<br>*Only common tests should be described solely by name; describe more complex techniques in the Methods section.*
☒	☐	A description of all covariates tested
☒	☐	A description of any assumptions or corrections, such as tests of normality and adjustment for multiple comparisons
☐	☒	A full description of the statistical parameters including central tendency (e.g. means) or other basic estimates (e.g. regression coefficient) AND variation (e.g. standard deviation) or associated estimates of uncertainty (e.g. confidence intervals)
☐	☒	For null hypothesis testing, the test statistic (e.g. F, t, r) with confidence intervals, effect sizes, degrees of freedom and P value noted<br>*Give P values as exact values whenever suitable.*
☒	☐	For Bayesian analysis, information on the choice of priors and Markov chain Monte Carlo settings
☒	☐	For hierarchical and complex designs, identification of the appropriate level for tests and full reporting of outcomes
☒	☐	Estimates of effect sizes (e.g. Cohen's d, Pearson's r), indicating how they were calculated

Our web collection on statistics for biologists contains articles on many of the points above.

Software and code

Policy information about availability of computer code

Data collection	IncuCyte S2 Live-Cell Analysis System (Essen Bioscience)<br>S360, Hamamatsu Slidescanner with Autoloader BD LSRFortessa<br>BD LSRFortessa
Data analysis	Graphpad Prism version 9<br>Flowjo version 10<br>BD FACSDiva version 9<br>OMERO.web version 5.22.1.

For manuscripts utilizing custom algorithms or software that are central to the research but not yet described in published literature, software must be made available to editors and reviewers. We strongly encourage code deposition in a community repository (e.g. GitHub). See the Nature Portfolio guidelines for submitting code & software for further information.

Data

Policy information about availability of data

All manuscripts must include a data availability statement. This statement should provide the following information, where applicable:

- Accession codes, unique identifiers, or web links for publicly available datasets
- A description of any restrictions on data availability
- For clinical datasets or third party data, please ensure that the statement adheres to our policy

All data are present in the article and supplementary information are available upon a reasonable request.

Research involving human participants, their data, or biological material

Policy information about studies with human participants or human data. See also policy information about sex, gender (identity/presentation), and sexual orientation and race, ethnicity and racism.

Reporting on sex and gender	N/A
Reporting on race, ethnicity, or other socially relevant groupings	N/A
Population characteristics	N/A
Recruitment	N/A
Ethics oversight	N/A

Note that full information on the approval of the study protocol must also be provided in the manuscript.

Field-specific reporting

Please select the one below that is the best fit for your research. If you are not sure, read the appropriate sections before making your selection.

☒ Life sciences ☐ Behavioural & social sciences ☐ Ecological, evolutionary & environmental sciences

For a reference copy of the document with all sections, see nature.com/documents/nr-reporting-summary-flat.pdf

Life sciences study design

All studies must disclose on these points even when the disclosure is negative.

Sample size	Sample sizes were indicated in the supplementary table 1 or in the figure legends. No power calculations were performed to predetermine sample size, and sample size were chosen based on experience.
Data exclusions	No data exclusions.
Replication	Biological replicates were used in all experiments. For cell culture experiments, results were obtained from one experiment in which three independent isolations of BMDMs for each genotype (n=3 biological replicates) were included.
Randomization	In vivo data collection from mice were randomized and selected based on mouse genotype.
Blinding	Investigators were only blinded while performing histological scoring. For the rest, the blinding was not possible as mouse genotype were screened to have necessary selection of mice.

Reporting for specific materials, systems and methods

We require information from authors about some types of materials, experimental systems and methods used in many studies. Here, indicate whether each material, system or method listed is relevant to your study. If you are not sure if a list item applies to your research, read the appropriate section before selecting a response.

Materials & experimental systems

n/a	Involved in the study	
	x	Antibodies
x		Eukaryotic cell lines
x		Palaeontology and archaeology
	x	Animals and other organisms
x		Clinical data
x		Dual use research of concern
x		Plants

Methods

n/a	Involved in the study	
x		ChIP-seq
	x	Flow cytometry
x		MRI-based neuroimaging

Antibodies

Antibodies used

Flow cytometry
anti-CD4 (eBioscience, cat no. 25-0041-81, clone: GK1.5, lot: 2123767, 1:200), anti-CD8 (eBioscience, cat no. 11-0081-82, clone: 53-6.7, lot:2002714, 1:100), anti-CD3e (eBioscience, cat no. 45-0031-82, clone: 145-2C11, lot:2284170, 1:200), anti-CD45 (eBioscience, cat no. 67-0451-82, clone: 30F11, lot:2551767, 1:200), anti-CD62L (eBioscience, cat no. 17-0621-82, clone: MEL-14, lot: 2213326, 1:200), anti-CD69 (eBioscience, cat no. 12-0691-82, clone:H1.2F3, lot:2468249, 1:200), anti-Ly6G (eBioscience, cat no. 46-9668-82, clone: 1A8, lot: 2093789, 1:100), anti-CD19 (eBioscience, cat no. 78-0193-82, clone: 1D3, lot: 1955736, 1:200), anti-CD11b (BD Horizon, cat no. 562317, clone: M1/70, lot:1132218, 1:100); anti-CD19 (eBioscience, cat no. 63-5941-82, clone:1D3, lot: 2366423, 1:100), anti-CD3e (eBioscience, cat no. 63-0031-82, clone:145-2c11, lot: 2205829, 1:100), anti-B220 (eBioscience, cat no. 63-0452-82, clone: RA36B2, lot: 2039913, 1:100), anti-NK1.1(eBioscience, cat no. 63-5941-82, clone: PK136, lot: 2348508, 1:100), anti-CD115 (BioLegend, cat no. 53-1152-82, clone: AFS98, lot: B283845, 1:50), anti-Ly6G cat no. 46-9668-82, clone: 1A8, lot:2093789, 1:100), anti-CD11b (BD Horizon, cat no. 562317, clone:M1/70, lot: 4339583, 1:100), anti-IL18Ra (Thermoscientific, cat no. 157903, clone:A17071D, lot: B367297, 1:50); anti-IL-33R (Biolegend, cat no. 145303, clone:DIH4, lot: B376782, 1:50), anti-CD3 (Invitrogen, cat no. HM3428, clone: 500A2, lot:2500513, 1:100), anti-Foxp3 (Thermoscientific, cat no. 12-5773-82, clone: FJK-165, lot:2176028, 1:20), anti-CD25 (BioLegend, cat no. 25-0251-82, clone: DC61.5, lot:2073783, 1:100), anti-CD4 (BioLegend, cat no. 17-0042-82, clone: RH4-5, lot: 2025255, 1:100).

Immunohistochemistry- All antibodies were diluted 1:500.
cleaved caspase-3 (CST, cat no. 9661, lot: 47), cleaved caspase-8 (CST, cat no. 8592, lot number:6); lysosome (Dako, cat no. F0372, lot: 00095833), CD45 (eBioscience, cat no.14-0451-82, clone: 30-F11, lot:2747801).

Western blot
p-IRF3 Ser396 (Thermo Scientific, cat no MA5-14947, clone: E.875.8, lot: YK4114271, 1:2000), IRF3 (CST, cat no. 4302, lot:7, 1:2000), IKKε (CST, cat no. 3416, lot:1, 1:2000), TBK1 (CST, cat no. 3013, lot:2, 1:2000), IL-1R1 (Santa Cruz, cat no sc-393998, clone:H-8, lot:E1622, 1:2000), GAPDH (Novus biologicals, cat. No NB300, clone:1D4, lot:021822, 1:10000), vinculin (CST, cat no. 13901, lot:7, 1:10000), alpha-tubulin (Sigma Aldrich, cat no. T6074, clone:B512, lot:YG374815, 1:10000).

Validation

All commerical available antibodies were validated by the manufacturer.

Animals and other research organisms

Policy information about studies involving animals; ARRIVE guidelines recommended for reporting animal research, and Sex and Gender in Research

Laboratory animals

Mice were housed in specific pathogen free enviroment at 22 ± 2 °C, with a humidity of 55 ± 10%, and an air exchange rate of 15 times per hour on a 12 h light-dark cycle. Mice are on C57BL/6 genetic background. Mice age is indicated in the figure legend and both sexes were used. The source of mice lines were indicated in the method section.

Wild animals

No wild animals were involved.

Reporting on sex

Sex was not considered in this study.

Field-collected samples

No samples were collected from the field.

Ethics oversight

All mouse procedures conducted in accordance with national and institutinal, and procols were approved by the responsible local authorities in Germany (Landesamt für Natur, Umwelt, und Verbraucherschutz Nordrhein-Westfalen).

Note that full information on the approval of the study protocol must also be provided in the manuscript.

Plants

Seed stocks	N/A
Novel plant genotypes	N/A
Authentication	N/A

Flow Cytometry

Plots

Confirm that:

- [ ] The axis labels state the marker and fluorochrome used (e.g. CD4-FITC).
- [ ] The axis scales are clearly visible. Include numbers along axes only for bottom left plot of group (a 'group' is an analysis of identical markers).
- [ ] All plots are contour plots with outliers or pseudocolor plots.
- [x] A numerical value for number of cells or percentage (with statistics) is provided.

Methodology

Sample preparation	Cells were harvested from organs flushing for bone marrow or by mechanical homogenization for spleen and thymus. Cell were filtered through a 70uM-cell strainer in flow cytometry buffer before staining.
Instrument	BD LSRFortessa
Software	BD FACSDiva v9 adn Flowjo v10
Cell population abundance	20000-50000 lie cells were acquired to measure abundance of target cell population.
Gating strategy	Gating strategy is provided in the Supplementray information.

- [x] Tick this box to confirm that a figure exemplifying the gating strategy is provided in the Supplementary Information.
